# Bioactive endophytic fungi isolated from Caesalpinia echinata Lam. (Brazilwood) and identification of beauvericin as a trypanocidal metabolite from Fusarium sp.

**DOI:** 10.1590/0074-02760140243

**Published:** 2015-02

**Authors:** Fernanda Fraga Campos, Policarpo A Sales, Alvaro José Romanha, Márcio SS Araújo, Ezequias P Siqueira, Jarbas M Resende, Tânia MA Alves, Olindo A Martins-Filho, Vera Lúcia dos Santos, Carlos A Rosa, Carlos L Zani, Betania Barros Cota

**Affiliations:** 1 Departamento de Ciências Biológicas e da Saúde, Universidade Federal dos Vales do Jequitinhonha e Mucuri, Diamantina, MG, Brasil; 2 Centro de Pesquisas René Rachou-Fiocruz, Belo Horizonte, MG, Brasil; 3 Departamento de Microbiologia, Imunologia e Parasitologia, Universidade Federal de Santa Catarina, Florianópolis, SC, Brasil; 4 Departamento de Química, Instituto de Ciências Exatas; 5 Departamento de Microbiologia, Instituto de Ciências Biológicas, Universidade Federal de Minas Gerais, Belo Horizonte, MG, Brasil

**Keywords:** endophytic fungi, bioactive, *Caesalpinia echinata* Lam., Fabaceae, *Trypanosoma cruzi*, beauvericin

## Abstract

Aiming to identify new sources of bioactive secondary metabolites, we isolated 82 endophytic fungi from stems and barks of the native Brazilian tree Caesalpinia echinata Lam. (Fabaceae). We tested their ethyl acetate extracts in several in vitro assays. The organic extracts from three isolates showed antibacterial activity against Staphylococcus aureus and Escherichia coli [minimal inhibitory concentration (MIC) 32-64 μg/mL]. One isolate inhibited the growth of Salmonella typhimurium (MIC 64 μg/mL) and two isolates inhibited the growth of Klebsiella oxytoca (MIC 64 μg/mL), Candida albicans and Candida tropicalis (MIC 64-128 μg/mL). Fourteen extracts at a concentration of 20 μg/mL showed antitumour activities against human breast cancer and human renal cancer cells, while two isolates showed anti-tumour activities against human melanoma cancer cells. Six extracts were able to reduce the proliferation of human peripheral blood mononuclear cells, indicating some degree of selective toxicity. Four isolates were able to inhibit Leishmania (Leishmania) amazonensis and one isolate inhibited Trypanosoma cruzi by at least 40% at 20 μg/mL. The trypanocidal extract obtained from Fusarium sp. [KF611679] culture was subjected to bioguided fractionation, which revealed beauvericin as the compound responsible for the observed toxicity of Fusarium sp. to T. cruzi. This depsipeptide showed a half maximal inhibitory concentration of 1.9 μg/mL (2.43 μM) in a T. cruzi cellular culture assay.

Neglected tropical diseases (NTDs) and cancer are disorders that generate a high global burden and novel therapies for these disorders are needed (Ehrenberg & Ault 2005, Farmer et al. 2010). Although a large number of antibiotics have saved hundreds of millions of lives over the last few years, the increase of opportunistic infections and antimicrobial resistance to drugs used in the clinic have contributed to the challenges faced by medicine in curing infectious diseases (Fauci & Morens 2012). The treatment of some cancers is palliative and this is not only a problem for the developed world (Farmer et al. 2010). The influence of small-molecules approved as drugs between 1981-2010, as natural products (N), and small-molecules directly derived from N (ND), is quite marked in the treatment of cancers (N = 11.1%, ND = 32.3%) and infectious diseases (N = 6.2%, ND = 40.9%) (Newman & Cragg 2012). NTDs, especially Chagas disease and leishmaniasis, affect poor and vulnerable groups and working to discover new medicines is not an attractive endeavour for pharmaceutical companies (Ehrenberg & Ault 2005). Several challenges in the treatment of these diseases, such as the ability of the drugs used clinically to cause toxic effects and their effectiveness at chronic stages, have not been overcome (Feasey et al. 2010).

The *Caesalpinia* genus (Leguminosae, Caesalpinioideae) includes approximately 130 species occurring in the tropics (Larsen et al. 1980, Lewis 1998). *Caesalpinia echinata* Lam. (Fabaceae) is an endangered species occurring in a highly threatened ecosystem. *C. echinata* is a native tree from Brazil that was the main source of red pigment in the XVI century during colonisation by Portugal and its popular name was given to the new land discovered when Portuguese navigators arrived in South America (Oliveira et al. 2002).

Endophytic fungi colonise all plant tissues (Petrini et al. 1992, Rodriguez et al. 2009, Vaz et al. 2009, Campos et al. 2011) and based on estimates, many fungal species and their secondary metabolites have not yet been described (Hyde & Soytong 2007, Hyde et al. 2007). The analysis of data recorded by PubMed and SciFinder in the last five years has revealed promising drug candidates from endophytic fungi that could be useful for different therapeutic applications.

Considering that only a small proportion of the existing endophytic fungi have been studied, especially those growing in tropical plants from Brazil, this paper focused on the investigation of the endophytic fungi living in the tissues of *C. echinata* as sources of bioactive natural products that could be used against some neglected diseases. In this work, we describe the taxonomic identification of the fungal isolates that produced biologically active extracts and the identification of beauvericin as the trypanocidal component of *Fusarium *sp. extract.

## subjects, Materials and Methods

*Plant material* - Healthy stems and barks of* C. echinata* were collected in Zoo-Botanical Foundation, Belo Horizonte (FZB-BH), state of Minas Gerais, Brazil, in March 2008. A voucher specimen was deposited at the FZB-BH Herbarium under the code BHZB-6458.

*Endophytic fungi isolation and storage* - Plant samples were collected in plastic bags and taken to the laboratory for processing. Plant material was washed in tap water, allowed to dry at room temperature (RT) and cut into pieces of approximately 1 × 1 cm. The surface of the fragments were sterilised by immersion in 70% ethanol (1 min) and 2% sodium hypochlorite (3 min), followed by one wash with sterile distilled water (2 min) (Collado et al. 1996). The fragments were plated onto potato dextrose agar (PDA) (Difco, USA) plates (Merck) containing 0.1 g/L chloramphenicol (Sigma, USA). The plates were incubated for up to 60 days at 25ºC and individual colonies were transferred to PDA. After complete growth, these colonies were photographed. Stock fungal cultures were deposited in the Culture Collection of Microorganisms and Cells of the Federal University of Minas Gerais. Fungal mycelial pieces were preserved at RT in sterile distilled water containing 30% v/v of glycerol.

*Cultivation and extraction of the fungal cultures* - Pieces of fungi mycelia (5 mm diameter) were transferred to five Petri dishes containing 20 mL of malt extract agar (PDA, Difco) medium (malt extract 1%, glucose 1%, peptone 0.1% and agar 1% in 1 L of purified water) and were cultured for 14 days at 28ºC. The biomass of the fungi mycelia were extracted by maceration with ethyl acetate for 48 h at RT. After passing through filter paper, the solvents were evaporated under reduced pressure using a rotary evaporator at 45ºC. Residual solvent in the extracts was eliminated in a vacuum centrifuge at 40ºC.

*Antimicrobial activity assays* - Antimicrobial activity was evaluated using the following microorganisms from the American Type Culture Collection (ATCC) (USA): *Staphylococcus aureus* ATCC 25295, *Escherichia coli* ATCC 18804, *Bacillus cereus* ATCC 11778, *Klebsiella oxytoca *ATCC 49131, *Salmonella typhimurium *ATCC 14028, *Pseudomonas aeruginosa* ATCC 27853, *Candida albicans *ATCC 18804 and* Candida tropicalis *ATCC 750.

Bacterial strains were maintained on brain heart infusion agar (Difco). Yeast strains were maintained on Sabouraud dextrose agar (Oxoid, UK).

*Culture media and inocula* - Mueller Hinton Broth (Himedia, India) was prepared in accordance with the Clinical and Laboratory Standards Institute (CLSI) document M7-A6 for minimal inhibitory concentration (MIC) bacterial assays (NCCLS 2003). Inocula of all bacteria were obtained using the spectrophotometric method prescribed by CLSI M7-A6, with final concentrations of 5 x 10^5 ^colony-forming unit (CFU)/mL. *Candida* cultures were grown at 35ºC and their inocula were prepared from fresh cultures according to the CLSI document M27-A2 (CLSI 2008). For the susceptibility tests, the final concentration was 1.5 x 10^3^ CFU/mL. After homogenisation by vortexing, the transmittance was measured at 520 nm and was adjusted to 69-70%.

*Susceptibility test* - The broth microdilution tests for bacteria and yeast were performed following the CLSI guidelines of M7-A6 and M27-A2, respectively. Susceptibility to antimicrobial agents was determined by the microbroth dilution method performed in sterile flat-bottom 96-well microplates (Difco). Fungal extracts were dissolved in dimethyl sulphoxide (DMSO) followed by the addition of Mueller Hinton Broth for bacterial assays and RPMI for yeast assays. Eight serial dilutions (2-256 µg/mL) were prepared using the corresponding media as the diluents and maintaining a constant volume of 1 mL in each tube. For each dilution, aliquots of 0.1 mL were distributed in the microplates. For growth and sterility control, media alone was used without the addition of extract and solvent. As a control for toxicity of the solvent, culture with DMSO was used. Chloramphenicol (Sigma-Aldrich) (0.78-100 µg/mL) was used as the positive antibacterial control and amphotericin B (AMB) (Sigma-Aldrich) (0.03-15 µg/mL) was used as the positive antifungal control. After the assembly of the plates, each bacterial and fungal strain was inoculated and the plates were incubated at 37ºC for 24 h for bacteria and 48 h for *Candida* species*.* Endpoints were determined visually by comparing the growth in the sample wells to the growth in drug-free control wells. MIC measurements were defined as the lowest sample concentration for which the well was optically clear and were expressed in µg/mL.

*Cytotoxicity assays with human cancer cell lines* - The assays were performed using the following tumour cell lines purchased from the National Cancer Institute (NCI) (USA): UACC-62 (human melanoma cancer), MCF-7 (human breast cancer) and TK-10 (human renal cancer). The cell toxicity assays were run according to the protocols established at NCI using the sulforhodamine colorimetric assay (Monks et al. 1991). Briefly, the cells were inoculated in 96-well plates and incubated at 37ºC for 24 h in a 5% CO_2_ atmosphere. The solutions of the test samples were added to the culture wells to attain the desired concentrations and the plates were incubated for another 48 h. Trichloroacetic acid was added to each well to precipitate the proteins, which were stained with sulforhodamine B. After washing out the unbound dye, the stained protein was dissolved in 10 mM Tris and absorbance was measured at the wavelength of 515 nm. The results were calculated using the absorbance measured in the test wells (T) in comparison with that of the control wells for the initial cell inoculum (Ti) and cells grown for 48 h without drug (Tf), using the following formula: [(T-Ti)/(Tf-Ti)] x 100. The results were expressed in terms of the growth inhibition percentage where the sample tested was considered cytostatic from 0-99% and cytocidal from 100-200%. Etoposide (ETO) at 1.6 µg/mL, culture medium without samples and culture medium with DMSO 1% (v/v) were used as controls.

*In vitro assay with human peripheral blood mononuclear cells (PBMC) *- *PBMC isolation from venous blood* - Venous blood from healthy adult volunteers was collected in heparinised tubes and centrifuged over a Ficoll-Hypaque cushion (Histopaque, Sigma). PBMC were collected from the Ficoll-Hypaque interphase and washed three times with RPMI-1640 medium (Gibco, USA). An aliquot of the cells was incubated with trypan blue (0.4% in NaCl 0.9%) and the viability of the cells was evaluated by visual inspection under a microscope. The cell suspensions were adjusted to 1.5 x 10^6^ cell/mL and cultured in RPMI-1640 medium supplemented with 5% (v/v) heat-inactivated, pooled human sera type AB (Flow Laboratories, Royaune-UNI) and L-glutamine (2 mM) (Gibco). An antibiotic/antimycotic solution containing 1 mg/mL penicillin, 1 mg/mL streptomycin and 25 µg/mL fungisone (Sigma) was added to control for fungal and bacterial contamination. In vitro cellular proliferation (blastogenesis) was assessed as previously described (Gazzinelli et al. 1983). Briefly, 1.5 x 10^5 ^cells were cultured in complete RPMI-1640 in flat-bottomed microtitre plates (Costar, tissue culture treated polystyrene # 3512, Sigma). The cultures were stimulated with 2.5 µg/mL of lectin from *Phaseolus vulgaris* phytohaemagglutinin (PHA) (Sigma) and incubated for 72 h at 37°C in a humidified atmosphere containing 5% CO_2_. Cell proliferation was determined using Alamar Blue according to the manufacturer's recommendations (Invitrogen, cat. DAL1100). The experiments were repeated three times using different samples of blood. Allopurinol and dexamethasone at 20 µg/mL were used as controls on PHA stimulated PBMC cultures. The results were expressed as percent inhibition of the PHA stimulated lymphocyte proliferation in relation to the control (no extracts added).

*In vitro assay with human PBMC* - The assay was performed as above, except that the culture was not stimulated with PHA. Cell toxicity was determined using Alamar Blue following the manufacturer's protocol (Invitrogen, cat. DAL1100). ETO at 20 µg/mL was used as the positive (toxic) control. The cytotoxic activity was evaluated by comparing the PBMC cultures with and without fungi extracts.

*Assays with Leishmania (Leishmania) amazonensis amastigotes-like forms* - Leishmanicidal activity was determined against amastigote-like forms of the parasite, which were obtained as previously described (Callahan et al. 1997). Briefly, promastigotes of *L. (L.) amazonensis* (strain IFLA/BR/196/PH-8) were obtained from lesions of infected hamsters. The parasites were grown at 26ºC in Schneider's medium (pH 7.2) and then stimulated to differentiate into amastigote forms by raising the temperature (32ºC) and lowering the pH (6.0) of the Schneider's medium. After seven days under these conditions, 90% of the promastigotes were transformed into amastigote-like forms, verified by microscopy and they were then used in the bioassays. The amastigote density was adjusted to 1 x 10^8^ parasites per mL and 90 μL was added to each well of the 96-well plates. Solutions of the test samples at 200 μg/mL containing DMSO 1% (v/v) in water were performed for each sample and then, 10 μL of the solution were added to each well of the 96-well plates. The plates were incubated at 32ºC for 72 h and then, cell viability was determined using the methyl thiazolyl tetrazolium assay (Teixeira et al. 2002). The results were calculated from the measured absorbencies using the formula [1-(Abs exp/Abs contr) x 100], which expresses the percentage of parasite death in relation to the controls without drug. AMB at 0.02 μg/mL (Fungison^(r)^, Bristol-Myers Squibb, Brazil) was used as a positive drug control.

*Assays with Trypanosoma cruzi amastigote and trypomastigote forms* - This assay was performed using *T. cruzi* (Tulahuen strain) expressing *E. coli β-*galactosidase as a reporter gene (Buckner et al. 1996, Romanha et al. 2010). Infective trypomastigote forms were obtained by monolayer culture of mouse L929 fibroblasts in RPMI-1640 medium (pH 7.2-7.4) without phenol red (Gibco) and with 10% foetal bovine serum and 2 mM glutamine. For the bioassay, 4,000 L929 cells in 80 μL of supplemented medium were added to each well of a 96-well microtitre plate. After an overnight incubation, 40,000 trypomastigotes in 20 μL were added to the cells and incubated for 2 h. The medium containing extracellular parasites was replaced with 200 μL of fresh medium and the plate was incubated for an additional 48 h to establish the infection. The medium was then replaced with solutions of the test samples at a concentration of 20 μg/mL in DMSO (< 1% in aqueous RPMI-1640 medium) and the plate was incubated for 96 h. To determine the half maximal inhibitory concentration (IC_50_) values, the cells were exposed to active samples at serial decreasing dilutions starting at 20 μg/mL and the IC_50_ values were calculated by linear interpolation. After this period, 50 μL of 500 μm chlorophenol red *β*-D-galactopyranoside in 0.5% Nonidet P-40 was added to each well and the plate was incubated for 16-20 h, after which the absorbance at 570 nm was measured. Controls with uninfected cells, untreated infected cells, infected cells treated with benznidazole (BNZ) at 1 μg/mL (positive control) and cells treated with DMSO 1% were used. Tetraplicates were run in the same plate and the experiments were repeated at least once.

The cytotoxicities of beauvericin and BNZ on uninfected mouse L929 fibroblasts were obtained. The IC_50_ values were calculated by linear interpolation and the selectivity index (SI) values were determined based on the ratio of the IC_50 _value in the host cell divided by the IC_50_ value of the parasite (Romanha et al. 2010).

*Statistical analysis* - The samples were tested in quadruplicate in the *T. cruzi *assays and in triplicate in the other assays. At least two independent experiments were performed. Values represent the mean ± variation coefficient.

*Molecular identification of endophytic fungi* - The extracts of 14 fungi showed positive results in at least one bioassay and thus were selected for molecular taxonomy. The DNA was extracted according to the procedure previously described (Rosa et al. 2009). The identification was based on the internal transcribed spacer-ribosomal DNA (ITS-rDNA) sequences. The pair of primers ITS1 (sequence: 5'-TCCGTAGGTGAACCTGCGG-3') and ITS4 (5'-TCCTCCGCTTATTGATATGC-3') was used for ITS-rDNA amplification (White et al. 1990). The sequences were generated using MEGABACE (Amersham Biosciences, USA), which were used to feed PHRED-PHRAP software to find the consensus sequence. The sequence was then compared with those deposited in GenBank using BLASTN software to identify the isolate down to the genus level. All fungal ITS-rDNA sequences obtained in this work were deposited in the GenBank with accessions KF611676-KF611689 (Table I).

**Table:** Table I Identification of endophytic fungi isolated from Caesalpinia echinata Lam. (Fabaceae) using primers internal transcribed spacer (ITS)1 and ITS4

WC	Closest related species	Similarity(%)	Base pairs analysed(n)	Identification and GenBankaccessions
25	*Aspergillus *sp*. *[KF367538.1]	100	523	*Aspergillus *sp. [KF611682]
45	*Epicoccum sorghi* [KC106717.1]	100	461	*E. sorghi* [KF611685]
46	*E. sorghi* [KC106698.1]	100	509	*E. sorghi* [KF611686]
9	*Fusarium *sp. [JQ905668.1]	100	357	*Fusarium* sp. [KF611679]
58	*Fusarium* sp. [HM631978.1]	99	481	*Fusarium* sp. [KF611688]
2	*Nectria pseudotrichia* [JN995626.1]	100	495	*N. pseudotrichia* [KF611677]
6	*N. pseudotrichia* [JF832647.1]	100	360	*N. pseudotrichia* [KF611678]
33	*N. pseudotrichia* [JN995626.1]	100	509	*N. pseudotrichia* [KF611683]
24	*Talaromyces* sp. [JX898040.1]	100	566	*Taralomyces* sp. [KF611681]
1	*Xylaria arbuscula *[JN601145.1]	99	497	*X. arbuscula* [KF611676]
11	*Xylaria* sp. [DQ322134.1]	96	515	*Xylaria* sp. [KF611680]
41	*Xylaria berteri *[JQ936300.1]	100	411	*X. berteri* [KF611684]
55	*Xylaria* sp. [JQ862693.1]	100	473	*Xylaria *sp. [KF611687]
84	*Xylaria *sp. [KC507252.1]	99	516	*Xylaria *sp. [KF611689]

WC: working code.

*Mass cultivation and extraction of Fusarium sp. [working code (WC) 9]* - Pieces of mycelia (5 mm diameter) were transferred to 30 Petri dishes containing 20 mL of PDA and were cultured for 14 days at 28ºC. The fungal biomass was placed in Erlenmeyer flasks and extracted by maceration with ethyl acetate for 48 h. The suspensions were filtered through filter paper and the filtrate evaporated to dryness under reduced pressure using a rotary evaporator at 45ºC. The residue was transferred to 20 mL flasks and the residual solvent was removed in a vacuum centrifuge at 40ºC for 18 h.

*Bioassay-guided fractionation of the Fusarium sp. extract* - The extract obtained (110 mg) was dissolved in 1 mL of a mixture of methanol (MeOH) and water (MeOH: H_2_O, 75:25 v/v). After centrifugation, the solution was injected into a semi-preparative reverse phase high-performance liquid chromatographic (RP-HPLC) column [250 mm × 4.6 mm internal dimension (i.d.)], 5 μm particle diameter) using a Shimadzu chromatograph (Shimadzu Corp, Japan) equipped with a LC6AD pump and manual injection valve (Rheodyne^TM^ 7125, Rheodyne Co, USA) using a fixed 1.000 µL sample loop and a dual-wavelength detector (SPD M10A) controlled by LCsolution software v.1.25 (Shimadzu Corp). The sample was purified with a linear gradient of water (A) and MeOH (B) using 10%B-100%B for 50 min and 100%B for 10 min. The eluent was pumped at 7 mL/min and the effluent absorption measured at λ 220 nm and 254 nm. Ten fractions corresponding to different peaks were collected and tested in the *T. cruzi* assay. Fraction (Fr)-5 (17 mg, 95% pure by HPLC) was the most active.

*Spectral data of the trypanocidal Fr-5* - Proton (^1^H) and carbon nuclear magnetic resonance (NMR) spectra, distortionless enhancement by polarisation transfer, heteronuclear single quantum coherence and heteronuclear multiple bond coherence (HMBC) experiments were performed on a Brucker DRX 400 spectrometer using the pulse programs provided by the manufacturer. The substance was dissolved in perdeuterated solvents doped with 0.1% tetramethyl silane as the internal standard.

Liquid chromatography-diode-array detection-mass spectrometry (LC-DAD-MS) analysis of Fr-5 was performed in a Thermo Surveyor Plus (Thermo Fisher Scientific, USA) chromatograph equipped with a Finnigan Surveyer PDA Plus diode-array detector and C18 column (Atlantis C18, Waters, USA) (3 μm particle diameter, 150 mm × 2.1 mm i.d.). A flow rate of 200 μL/min was used and the effluent entirely directed the Bruker ETD-maXis quadrupole TOF (Bruker Daltonics, Germany) for electrospray ionisation (ESI) in the positive ion mode. The LC-DAD-MS was conducted in a gradient system using a mixture of water (A) and MeOH (B) with 0.1% formic acid, 1%B-100%B for 13 min, 100%B for 4 min, 100%B-1%B for 0.5 min, 1%B for 11.5 min.

The mass detector was set to the mass-to-charge ratio (*m/z*) range of 50-1500 atomic mass units. The instrument was operated under the following conditions: end plate offset, -500 voltage (V); capillary V, 4.500 V; nebuliser pressure, 0.4 bar; dry gas (nitrogen) flow rate, 4.0 L/min; dry temperature, 180ºC; collision-induced dissociation energy, 25 eV; collision energy, 7 eV; ion cooler radio-frequency, 25 excitation V; transfer time, 45 μs.

Fr-5 (beauvericin): white powder; specific optical rotation = + 47 [concentration at g/100 mL (c) 0.8, MeOH]; ultraviolet (UV) (DAD, MeOH) maximum wavelength (λ_max_) 202 nm. ^1^H NMR [deuterated MeOH (CD_3_OD), 400 megahertz (MHz)] chemical dislocation in ppm (*δ*)_H_ 7.26-7.24 [12H, multiplet (m), H-10/H-11/H-13/H-14], 7.17 (3H, m, H-12), 5.45 [3H, doublet of doublets (dd), coupling constant (*J*) = 10.9 and 3.9, Hz, H-7], 4.92 [3H, dublet (d), *J* = 8.6 Hz, H-1], 3.36 (3H, dd, *J* = 14.5 and 5.0 Hz, H-8b), 2.99 [9H, singlet (s), H-6], 2.97 (3H, dd, *J* = 11.8 Hz, H-8a), 2.01 (1H, td, *J* = 21.2, 6.8 and 6.7 Hz, H-2), 0.78 (9H, d, *J* = 6.6 Hz, H-3), 0.43 (9H, d, *J* = 6.7 Hz, H-4). Carbon-13 ^(13^C) NMR (CD_3_OD, 100 MHz): *δ*_C_ 170.2 (C, C-5), 169.7 (C, C-15), 136.9 (C, C-9), 129.1 and 128.8 (CH, C-10/C-11/C-13/C-14), 127.0 (CH, C-12), 75.7 (CH, C-1), 57.6 (CH, C-7), 35.0 (CH_2_, C-8), 32.6 (CH_3_, C-6), 29.9 (CH, C-2), 18.5 (CH_3_, C-3), 17.7 (CH_3_, C-4); high resolution-ESI-MS *m*/*z *784.4180 [M + H]^+^ (calcd. for C_45_H_57_N_3_O_9_, 783.4095).

*High performance LC coupled to an UV detector (HPLC-DAD) analysis* - The ethyl acetate extract from *Fusarium *sp. (WC 9) and Fr-5 (beauvericin) were analysed by HPLC-DAD in a Shimadzu chromatograph (Shimadzu Corp) equipped with a LC10AD pump and manual injection valve (Rheodyne^TM^ 7725i, Rheodyne Co) using a fixed 20 µL sample loop, a CTO-20A thermostat-controlled oven compartment and a SPD-M20A diode array detector (190-800 nm) controlled by LCsolution software. A Shim-pack^(r)^ C18 column (5 μm, 250 mm × 4.6 mm i.d.) maintained at 40ºC was used in the chromatographic analysis. The separations were conducted in a gradient system, using a mixture of water (A) and acetonitrile (ACN) (B) with 0.1% trifluoroacetic acid (TFA), 10%B-100%B for 30 min and 100%B for 10 min as the mobile phase, at a flow rate of 1.0 mL/min. The ethyl acetate extract (5 mg/mL) and Fr-5 (beauvericin) (200 µg/mL) were dissolved in MeOH. The particulates were removed by centrifugation and the sample injection volume was 20 μL for each sample.

## Results

*Isolation and molecular identification of endophytic fungi from C. echinata Lam.* - Eighty-two endophytic fungal strains were isolated from plant bark (10 samples) and stems (13 samples) (Table II). Fourteen fungal isolates yielded extracts that were active in vitro at 20 μg/mL in at least one biological assay. Based on the results of their ITS1-5.8S-ITS4 partial sequences, these isolates were submitted to the GenBank to obtain their accession numbers and the closest related species were achieved by BLAST analysis. The results (Table I) show that all sequences had more than 96% similarity with the species in GenBank. Most sequences presented 99% (n = 3) or 100% (n = 10) similarity to the closest related species in GenBank.

**Table:** Table II Number of isolates from different plant parts

Plant part	Samples(n)	Fungi isolated(n)	Morphotypes(n)
Barks	10	34	14
Stems	13	48	10
Total	-	82	-

All 14 isolates belong to the Ascomycota phylum, with 10 from the Sordariomycetes class, two from the Eurotiomycetes class and two from the Dothideomycetes class. Among the fungi that produced active extracts, five were from the *Xylaria* genus (35%) and three were from the *Nectria* genus (21%). *Fusarium* and *Epicoccum *afforded two active extracts each (14%) and* Taralomyces* and *Aspergillus* afforded one active extract each (7%) (Tables III, IV).

*Biological activities of extracts from endophytic fungi isolated of C. echinata Lam.* - The ethyl acetate extracts of 82 fungal isolates from *C. echinata Lam.* were tested in in vitro biological assays to predict their leishmanicidal, trypanocidal, cytotoxic and antimicrobial activities. Forty-four fungal isolates were considered active (≥ 40% of the inhibition of growth) in at least one biological assay (Tables III, IV).

The extracts from *Talaromyces* sp. (WC 24), *Aspergillus *sp. (WC 25) and *Epicoccum sorghi* (WC 45) showed antibacterial activity against Gram-positive and Gram-negative bacterial species (*S. aureus* and *E. coli*) with MIC values ranging from 32-64 μg/mL. *Aspergillus *sp. extract (WC 25) showed an MIC value of 64 μg/mL against *S. typhimurium *and* K. oxytoca. Talaromyces *sp. extract (WC 24) showed an MIC of 64 μg/mL against *K. oxytoca*. Antifungal activity was only observed for the extracts from *Fusarium* sp. (WC 9) and *Nectria pseudotrichia *(WC 33), which inhibited *C. albicans *and* C. tropicalis *at concentrations ranging from 64-128 μg/mL (Table III).

**Table:** Table III In vitro antimicrobial activities of extracts from endophytic fungi of Caesalpinia echinata Lam. (Fabaceae)

Fungal isolate (WC)	Microorganisms							
	Minimal inhibitory concentration (MIC) (µg/mL)							
	SA	EC	BC	ST	PA	KO	CA	CT
*Aspergillus *sp. (25)	32	64	> 256	64	> 256	64	> 256	> 256
*Epicoccum sorghi* (45)	64	32	> 256	> 256	> 256	> 256	> 256	> 256
*E. sorghi* (46)	> 256	> 256	> 256	> 256	> 256	> 256	> 256	> 256
*Fusarium* sp. (9)	> 256	> 256	> 256	> 256	> 256	> 256	64	128
*Fusarium *sp. (58)	> 256	> 256	> 256	> 256	> 256	> 256	> 256	> 256
*Nectria pseudotrichia *(2)	> 256	> 256	> 256	> 256	> 256	> 256	> 256	> 256
*N. pseudotrichia *(6)	> 256	> 256	> 256	> 256	> 256	> 256	> 256	> 256
*N. pseudotrichia *(33)	64	> 256	> 256	> 256	> 256	> 256	128	128
*Talaromyces* sp. (24)	32	64	> 256	> 256	> 256	64	> 256	> 256
*Xylaria arbuscula *(1)	> 256	> 256	> 256	> 256	> 256	> 256	> 256	> 256
*Xylaria* sp. (11)	> 256	> 256	> 256	> 256	> 256	> 256	> 256	> 256
*Xylaria berteri *(41)	> 256	> 256	> 256	> 256	> 256	> 256	> 256	> 256
*Xylaria *sp.**(55)	> 256	> 256	> 256	> 256	> 256	> 256	> 256	> 256
*Xylaria *sp.**(84)	64	> 256	> 256	> 256	> 256	> 256	> 256	> 256
Controls								
Amphotericin B	NT	NT	NT	NT	NT	NT	0.12	1.2
Chloramphenicol	16	8	16	16	8	8	NT	NT

BC: *Bacillus cereus*; CA: *Candida albicans*; CT: *Candida tropicalis*; EC: *Escherichia coli*; KO: *Klebsiella oxytoca*; NT: not tested; PA: *Pseudomonas aeruginosa*; SA: *Staphylococcus aureus*; ST: *Salmonella typhimurium*; WC: working code. Values in bold mean extracts with MIC values = 128 µg/mL.

Four isolates, *Fusarium* sp. (WC 9),* Xylaria* sp. (WC 11),* N. pseudotrichia *(WC 33) and *Fusarium* sp. (WC 58), were able to inhibit the growth (45-77%) of the amastigote forms of *L. (L.) amazonensis*. However, only *Fusarium* sp. (WC 9) inhibited (92%) the amastigote and trypomastigote forms of *T. cruzi* when tested at 20 μg/mL (Table IV)*.*

**Table:** Table IV In vitro antiprotozoan, cytotoxic and antiproliferative activities of extracts from endophytic fungi of Caesalpinia echinata Lam. (Fabaceae)

Fungal isolate**(WC)	Tumour cell lineages(%)				PBMC(%)			Protozoan(%)	
	UACC-62	TK-10	MCF-7		Mortality	Proliferation decreased		LA	TC
*Aspergillus *sp.**(25)	-	103 ± 9	-		-	-		-	-
*Epicoccum sorghi* (45)	75 ± 7	47 ± 10	67 ± 9		-	-		-	-
*E. sorghi* (46)	-	97 ± 5	60 ± 17		-	15 ± 7		-	-
*Fusarium* sp. (9)	-	98 ± 5	88 ± 4		-	15 ± 8		45 ± 0	92 ± 4
*Fusarium *sp.**(58)	-	-	48 ± 10		49 ± 26	-		45± 4	-
*Nectria pseudotrichia *(2)	-	54 ± 14	68 ± 4		NT	NT		-	-
*N. pseudotrichia *(6)	-	95 ± 7	93 ± 0		NT	NT		-	-
*N. pseudotrichia *(33)	102 ± 7	96 ± 15	60 ± 11		-	37 ± 15		77 ± 3	-
*Talaromyces* sp. (24)	-	95 ± 9	60 ± 7		-	20 ± 13		-	-
*Xylaria arbuscula *(1)	-	60 ± 1	57 ± 9		-	-		-	-
*Xylaria* sp. (11)	-	113 ± 3	47 ± 8		NT	NT		51 ± 1	-
*Xylaria berteri *(41)	-	43± 6	-		-	20 ± 15		-	-
*Xylaria *sp.**(55)	-	92 ± 4	58 ± 0		-	-		-	-
*Xylaria *sp.**(84)	-	51±9	48 ± 8		NT	NT		-	-
Controls									
AMB	NT	NT	NT		NT	NT		82 ± 3	NT
BNZ	NT	NT	NT		NT	NT		NT	86 ± 8
ETO	176 ± 9	185 ± 9	100 ± 5		33 ± 14	NT		NT	NT
DEX	NT	NT	NT		18 ± 13	-		NT	NT
ALL	NT	NT	NT		-	21 ± 14		NT	NT

all extracts were tested at 20 µg/mL. Results were expressed in terms of percentage of the inhibition. ALL: allopurinol tested at 20 µg/mL; AMB: amphotericin B tested at 0.02 µg/mL; BNZ: benznidazole tested at 1.0 µg/mL = 3.8 µM; DEX: dexamethasone tested at 20 µg/mL; ETO: etoposide tested at 1.6 µg/mL in tumour cell lineages and at 20 µg/mL in human peripheral blood mononuclear cell (PBMC); LA: amastigotes forms of *Leishmania (Leishmania) amazonensis*; MCF-7: human breast cancer; NT: not tested; TC: amastigote and trypomastigote forms of *Trypanosoma cruzi*; TK-10: human renal cancer; UACC-62: human melanoma cancer; WC: working code; -: inactive;

Fourteen fungal isolates exhibited cytotoxicity toward MCF-7 and TK-10 cell lineages, inhibiting their growth by at least 40%. In addition, the extracts of *N. pseudotrichia* (WC 33) and *E. sorghi* (WC 45) inhibited the growth of UACC-62. The extracts of tree fungi (*Xylaria* sp., WC 11; *Aspergillus *sp. WC 25 and *N. pseudotrichia*, WC 33) displayed cytocidal activity at 20 μg/mL; in other words, the number of viable cells was less than in the initial inoculum (Table IV). Although all 14 isolates showed some degree of cytotoxicity against three tumour cell lineages at 20 μg/mL, only one was cytotoxic to human PBMCs and six were able to reduce the PHA stimulated proliferation of PBMCs (Table IV).

**Fig. 1:**
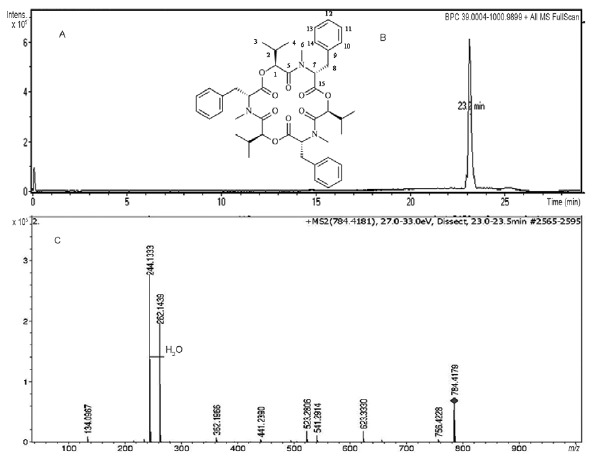
total-ion chromatogram (A) of beauvericin (B). Column RP-18, 150 mm × 2.1 mm i.d.; mobile phase [A: H2O; B: methanol) with 0.1% formic acid; 1%B-100%B in 13 min, 100%B in 4 min, 100%B-1%B in 0.5 min, 1%B in 11.5 min flow rate 200 µL/min-1. Electrospray ionisation-(+)-MS/MS of (B) (precursor m/z 784.4179 [M + H]+) and main fragments (C).

*Chemical characterisation of the trypanocidal compound of the Fusarium sp. extract and HPLC-DAD analysis* - To identify the trypanocidal component of *Fusa- rium* sp. (WC 9), the ethyl acetate extract from this strain was produced at a larger amount and was submitted to semi-preparative RP-HPLC fractionation. The fractions were tested in the intracellular *T. cruzi* assay and only Fr-5 was able to kill 100% of the *T. cruzi* amastigote and trypomastigote forms at a concentration of 5 μg/mL. The HPLC chromatogram of Fr-5 showed a single peak (Fig. 1A) with UV purity index of approximately 95%. The spectral data of Fr-5 were in full agreement with those reported for beauvericin (Fig. 1B) (Newman 2008, Hu & Rychlik 2012). The UV spectra exhibited absorptions between 196-230 nm (λ_max _202 nm), which is consistent with previous reports (Monti et al. 2000, Mahnine et al. 2011). The HRMS of Fr-5 showed a *quasi*-molecular [M + H]^+^ ion peak at *m*/*z *784.4180, which is consistent with the molecular formula of: C_45_H_57_N_3_O_9_ (calcd. 783.4095) (Fig. 1C). The ^13^C NMR spectrum showed 15 carbon signals, which together with the mass spectra analysis suggested a symmetrical structure for Fr-5. The ^1^H and ^13^C NMR spectra of Fr-5 showed signals of aromatic carbons and ^1^H (δ_H_ 7.26-7.17, δ_C_ 127.0-129.1) from a benzyl moiety (δ_H_ 3.36, dd, *J* = 14.5 and 5.0 Hz/δ_C_ 35.0) due to the phenylalanine residues. The ESI-(+)-LC-MS/MS spectra of Fr-5 (Fig. 1C) showed fragment ions at *m/z* 262.1439 and 244.1324 produced by the cleavage of the phenylalanine amide bond followed by loss of H_2_O, as observed in beauvericin (Sewram et al. 1999, Hu & Rychlik 2012). The hydroxy-isovaleryl moiety showed ^1^H signals at δ_H_ 4.92 (1H, d, *J* = 8.6 Hz/ δ_C_ 75.7), δ_H_ 2.01 (1H, td, *J* = 21.2, 6.8 and 6.7 Hz/ δ_C_ 29.9), δ_H_ 0.78 (3H, d, *J* = 6.6 Hz/δ_C_ 18.5) and δ_H_ 0.43 (3H, d, *J* = 6.7 Hz/δ_C_ 17.7). In addition, the *N*-methylamino acid moiety of beauvericin matches the signals of Fr-5 at δ 2.99 (3H, s, δ_C_ 32.6, N-CH_3_).

**Fig. 2:**
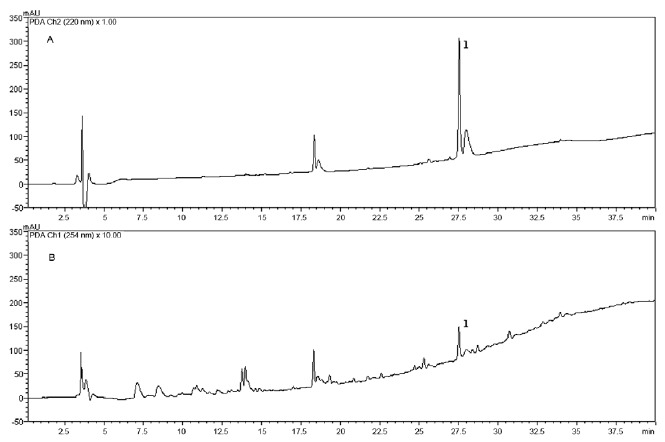
high performance liquid chromatographic coupled to an ultraviolet (UV) detector profile of ethyl acetate Fusarium sp. (working code 9). UV detection at 220 nm (A) and 254 nm (B). Column RP-18, 250 mm × 4.6 mm i.d.; mobile phase (A: H2O; B: acetonitrile) with 0.1% trifluoroacetic acid; 10%B-100%B in 30 min, 100%B in 10 min, flow rate of 1.0 mL/min. The ethyl acetate extract (5 mg/mL) and beauvericin (1,200 µg/mL).

The ethyl acetate extract from *Fusarium *sp. (WC 9) was analysed using RP-HPLC-DAD and beauvericin (1) eluted after 27.5 min (Fig. 2) using a mixture of ACN and water with 0.1% TFA.

*Trypanocidal and cytotoxicity activities of the beauvericin* - While the crude extract of *Fusarium* sp. WC 9 showed an IC_50_ of 30 μg/mL in the assay with *T. cruzi* forms expressing the *β*-galactosidase gene, Fr-5 (beauvericin) showed an IC_50_ value 15 times smaller (1.9 μg/mL, 2.43 μM). This compound showed cytotoxic activity against the host cell (mouse L929 fibroblasts) used in the *T. cruzi* assay, showing an IC_50_ of 5 μg/mL (6.38 μM). Thus, under our assay conditions, beauvericin showed an SI of only 2.7. BNZ was used as the standard and showed an IC_50_ value of 3.8 μM and an SI value of 625 against mouse L929 fibroblasts.

## Discussion

In our previous work (Cota et al. 2011), we found that the crude ethanol extract of *C. echinata* Lam. kills 90% of the amastigote-like forms of *L. amazonensis *at a concentration of 20 µg/mL. This promising result prompted us to continue to study *C. echinata* as a host plant of potential bioactive endophytic fungi. Previous work by other groups identified 43 taxa belonging to Hyphomycetes and three belonging to Coelomycetes in leaf litter of *C. echinata* Lam. (Grandi & Silva 2003, 2006). They reported the presence of *Epicoccum nigrum* as an anamorphic fungi (Grandi & Silva 2006). In the present study, we identified two isolates (*E. sorghi*; WC 45 and 46) of the same genus as bioactive endophytic fungi.

Few reports are available on the biological activities of fungi growing in* C. echinata.* Machado (2009) isolated *Botryosphaeria rhodina, Xylaria multiplex *and* Pestalotiopsis *sp. as endophytic fungi from the leaves and stems of *C. echinata*. Although none of these isolates were active against *Enterococcus faecalis, P. aeruginosa *or* S. aureus* by agar diffusion assay (100 μg and 1,000 μg), they were able to inhibit the growth of the phytopathogens *Pythium debaryanum* and *Phytoththora palmivora *(Machado 2009).

Most of the fungi identified in the present work have been previously reported as endophytic in other plants. *Xylaria*, *Nectria* and *Aspergillus* genera are found in *Piper aduncum* (Piperaceae) and many other plants in Brazilian savannas (Martínez-Luis et al. 2011, Vaz et al. 2012). In addition, *Fusarium* species are the most frequent endophytes (Liang et al. 2012). The other two genera described in this paper, *Taralomyces* and *Epicoccum,* were recently found to be endophytic (Fávaro et al. 2012, Bara et al. 2013).

Our results show that, overall, approximately 17% of our fungal isolates were active in at least one of the four bioassays performed. Recently, Higginbotham et al. (2013) showed that fungi isolated from the plant family Fabaceae (Fabales) had a high percentage of highly active genotypes and were associated with moderate activity against *Plasmodium falciparum* and MCF-7 cells (breast cancer cell line) when tested at a concentration of 10 µg/mL. Moreover, extracts from fungi of *Aspergillus* and *Xylaria *genera are the most active endophytic fungi according to the results of in vitro assays of these fungi against *Leishmania donovani*, *T. cruzi* and MCF-7 cells (Higginbotham et al. 2013). The results of our biological assays lead us believe that all isolates tested, except for the isolate *Fusarium* sp. (WC 58), which showed high toxicity to the PBMC in vitro, are potential sources of compounds useful in the development of drugs against infectious agents and immunomodulatory metabolites.

In the present study, the fungi *Fusarium *sp. [KF611679] was the only one that showed activity against *T. cruzi* amastigote and trypomastigote forms and exhibited the best MIC values against *C*. *albicans* and *C*.* tropicalis*. Several *Fusarium* species isolated from plants are known to produce secondary metabolites, such as terpenoids, alkaloids and mycotoxins, with promising biological activities (Hyde & Soytong 2007, Hyde et al. 2007, Campos et al. 2012).

The trypanocidal activity of the fungi extract [KF611679] was attributed to beauvericin. Beauvericin is a mycotoxin produced by many fungi, including *Fusarium *spp (Wang & Xu 2012). Beauvericin displays insecticidal (Hamill et al. 1969), antitumour (Cheng et al. 2009), antibacterial, antifungal and antiviral activities (Zhan et al. 2007, Shin et al. 2009, Meca et al. 2010, Xu et al. 2010). Beauvericin was also reported to have leishmanicidal activity (EC_50_ 1.86 μM) against promastigotes of *Leishmania braziliensis *(Nascimento et al. 2012). To the best of our knowledge, this is the first report on the trypanocidal activity of this cyclic hexadepsipeptide. Our results support those of previous studies (Klarić et al. 2008, Nascimento et al. 2012) that also showed that this compound was cytotoxic; we obtained an IC_50_ of 5 μg/mL (6.38 μM) against the host cell (mouse L929 fibroblasts) used in a *T. cruzi* assay. Under our assay conditions, beauvericin showed an SI of only 2.7, a value that, according to current guidelines (Romanha et al. 2010), is too low for beauvericin to be considered for pre-clinical studies.

Notwithstanding, according to a recent review (Feudjio et al. 2010), beauvericin-mediated cytotoxicity towards various mammalian and cancer cell lines is only partially understood and involves several cellular targets and molecular mechanisms. Furthermore, only a few studies have addressed the effects of beauvericin in animals and those studies have found only minor acute toxic effects. The authors emphasised that the consequences of chronic exposure and of pharmacologically active doses of beauvericin in humans/animals have not been explored in detail. Therefore, the biological activities of beauvericin on mammalian cancer cells and protozoan parasites suggest that beauvericin is a potential drug candidate for the treatment of cancers and infectious diseases. There is a need for further studies to determine the efficacy and safety of beauvericin in animals infected with *T. cruzi*.

In conclusion, this work demonstrated the in vitro leishmanicidal, trypanocidal, antimicrobial and cytotoxic activities of crude extracts prepared from endophytic fungi isolated from stems and barks of *C. echinata*. In addition, the bioassay-guided fractionation of *Fusarium* sp. (WC 9) extract using the *T. cruzi* assay allowed us to identify the cyclic hexadepsipeptide mycotoxin beauvericin as the trypanocidal component produced by the fungus.
